# Selection and Characterization of Cell Line–Virus Pairs for Sensitive Viral Detection Assays in Biopharmaceutical Testing

**DOI:** 10.3390/mps9040107

**Published:** 2026-07-08

**Authors:** Agnieszka Staniszewska, Agnieszka Piastowska-Ciesielska

**Affiliations:** Department of Cell Cultures and Genomic Analysis, Medical University of Lodz, 90-752 Lodz, Poland

**Keywords:** viral safety, adventitious agents, cell line–virus pairing, cytopathic effect (CPE), TCID_50_, limit of quantification (LOQ), biopharmaceuticals, quality control, in vitro assay, Vero cells, MRC-5, BHK-21, Reovirus type 3, Adenovirus type 5, human parainfluenza virus type 3, herpes simplex virus

## Abstract

Ensuring viral safety is a critical aspect of biopharmaceutical production, requiring sensitive and reliable methods for detecting adventitious agents. In this study, we systematically evaluated the performance of selected cell line–virus combinations to identify optimal models for in vitro viral detection assays. Three cell lines (Vero, MRC-5 and BHK-21 [C-13]) and representative model viruses (Reovirus type 3, Adenovirus type 5, Human parainfluenza virus type 3, and Herpes simplex virus) were analyzed in terms of cytopathic effect (CPE) kinetics, morphology, and detection sensitivity. All tested systems demonstrated high analytical sensitivity, with limits of quantification (LOQ) reaching 0.01 TCID_50_/mL for selected viruses. However, substantial differences were observed in infection dynamics and CPE morphology depending on the cell line–virus combination. BHK-21 [C-13] cells exhibited the most rapid and pronounced CPE for Reovirus type 3, enabling early and unambiguous detection. Vero cells provided robust and reproducible detection of Adenovirus type 5, characterized by well-defined cytopathic progression. MRC-5 cells showed controlled and consistent infection kinetics for both Human parainfluenza virus type 3 and Herpes simplex virus, allowing improved temporal resolution and interpretability. These findings demonstrate that assay performance depends not only on sensitivity but also on the kinetics and morphology of infection. Based on combined evaluation criteria, the following optimal cell line–virus pairs were identified: BHK-21 [C-13]/Reovirus type 3, Vero/Adenovirus type 5, and MRC-5/Human parainfluenza virus type 3 and Herpes simplex virus. The proposed approach supports rational selection of detection models and provides a preliminary descriptive framework for the development of routine visual screening assays in biopharmaceutical quality control.

## 1. Introduction

Biopharmaceuticals made in mammalian cells (especially Chinese Hamster Ovary (CHO) cells) carry an inherent risk of adventitious viral contamination, mainly from raw materials and cell substrates. Despite this, modern viral safety strategies have made clinical transmission to patients extremely rare. Contamination events are rare relative to the huge number of batches, but when they occur, they can shut down facilities, cost tens of millions of dollars, and cause drug shortages [1,2,3,4]. Most documented events in CHO-derived products involved mouse minute virus, reovirus, Cache Valley virus, epizootic hemorrhagic disease virus, and vesivirus 2117, usually traced to raw materials like animal serum or media components [1,3,4,5]. Cell lines themselves can be contaminated (e.g., transformed lines producing virus), posing risks to operators and products [4,6]. In vitro assays using permissive cell lines remain the gold standard for detecting viral, bacterial, and mycoplasma contaminants, typically relying on cytopathic effect (CPE) or infectivity-based endpoints such as TCID_50_. These methods are complemented by molecular techniques like PCR and next-generation sequencing (NGS), which offer increased sensitivity and specificity but may not always indicate the presence of infectious agents [7]. Traditional vitro assays involve culturing test samples on panels of indicator cell lines (e.g., Vero, CHO, MRC-5) and monitoring for CPE or other infection markers such as hemadsorption or hemagglutination. Regulatory guidelines (e.g., ICH Q5A) recommend using multiple cell lines to maximize detection breadth, systematic evaluations of cell line–virus pairings and their impact on assay sensitivity need to be done before method implementation [5,8,9]. Systematic studies show that standard in vitro assays can detect a wide range of viruses at low titers—often below 1 TCID_50_ or PFU—when appropriate cell lines are used. However, detection limits vary depending on the virus-cell line combination; some viruses require special conditions or exhibit low sensitivity. The use of multiple cell lines increases the likelihood of detecting diverse contaminants [5,8,10]. The aim of this study was to systematically evaluate and characterize selected cell line–virus pairs in order to identify optimal combinations for sensitive and reproducible viral detection assays applicable to biopharmaceutical testing. Particular emphasis was placed on determining the limit of quantification (LOQ), analyzing cytopathic effect kinetics, and assessing the suitability of selected models for implementation in quality control environments. To establish a proof-of-concept framework for assessing pathogen-host system susceptibility, this study evaluates four distinct viral models representing diverse structural and physicochemical properties: Reovirus type 3 (Reo-3), Adenovirus type 5 (Ad5), Human parainfluenza virus type 3 (HPIV-3), and Herpes simplex virus type 1 (HSV-1). These agents reflect a broad matrix of characteristics (DNA vs. RNA, enveloped vs. non-enveloped) frequently highlighted in regulatory guidelines, such as ICH Q5A, for viral safety testing in biopharmaceutical production. From a pathogen-entry perspective, their cell tropism is mediated by distinct surface receptors—Ad5 utilizes the Coxsackievirus and Adenovirus Receptor (CAR), HSV-1 binds to nectin-1/HVEM, Reo-3 targets sialic acid and JAM-A, while HPIV-3 engages sialic acid receptors. Evaluating these diverse replication mechanisms across CHO, Vero, MRC-5, and BHK-21 lines provides a comprehensive reference for screening assay sensitivity.

## 2. Materials and Methods

### 2.1. Cell Lines

The following mammalian cell lines were used in this study: Vero (ATCC, Manassas, VA, USA), MRC-5 (ATCC, Manassas, VA, USA), and BHK-21 [C-13] (ATCC, Manassas, VA, USA). Cell lines were selected to represent different host origins and susceptibility profiles relevant for viral safety testing in biopharmaceutical applications. Cells were maintained in Eagle’s Minimum Essential Medium (EMEM; ATCC, Manassas, VA, USA), supplemented with 10% fetal bovine serum (FBS; Gibco, Thermo Fisher Scientific, Grand Island, NY, USA), 1% L-glutamine (Gibco, Thermo Fisher Scientific, Grand Island, NY, USA), and antibiotics (penicillin/streptomycin; Gibco, Thermo Fisher Scientific, Grand Island, NY, USA). Cells were incubated at 37 °C in a humidified atmosphere containing 5% CO_2_.

### 2.2. Virus Stocks

The following model viruses were used for infection experiments: Human parainfluenza virus type 3 (HPIV-3; ATCC, Manassas, VA, USA), Herpes simplex virus (HSV-1; ATCC, Manassas, VA, USA), Adenovirus 5 (Ad5; ATCC, Manassas, VA, USA) and Reovirus 3 (Reo-3; ATCC, Manassas, VA, USA). These viruses were selected based on their distinct biological properties, including genome type, presence of viral envelopes, and replication kinetics in mammalian cell systems.

Viral stocks were propagated in permissive cell lines under standard culture conditions. Following infection, supernatants were harvested after observation of complete cytopathic effect (CPE), clarified by low-speed centrifugation, and stored at −80 °C until further use.

### 2.3. Virus Titration (TCID_50_)

Viral titers were determined using the median tissue culture infectious dose (TCID_50_) assay based on the Reed and Muench method. Briefly, serial ten-fold dilutions of viral stocks were prepared and inoculated onto confluent monolayers of appropriate cell lines in 96-well plates. Plates were incubated under standard conditions and monitored for the presence of cytopathic effect over a defined observation period. Viral titers were calculated as TCID_50_/mL.

### 2.4. Infection Assay (CPE Assay)

For infection experiments, cells were seeded in T25 flaks (Thermo Fisher Scientific, Waltham, MA, USA) at densities optimized for each cell line to achieve 70–80% confluency at the time of infection. After adherence, cells were infected with serial dilutions of virus ranging from 10 to 0.01 TCID_50_/mL.

Following infection, flasks were incubated under standard culture conditions and observed daily for cytopathic effect (CPE). CPE was assessed microscopically based on morphological changes including cell rounding, detachment, and monolayer disruption. The time to onset and progression of CPE were recorded for each cell line–virus combination.

In parallel, mock-infected controls (cells replenished with maintenance medium without virus) were maintained under identical conditions for each cell line to serve as a baseline for normal cell morphology and monolayer integrity.

### 2.5. Determination of LOQ

The limit of quantification (LOQ) was defined as the lowest viral concentration at which a reproducible cytopathic effect was observed in 100% of replicate flasks across independent experiments.

### 2.6. Experimental Design for Cell-Virus Pairing Evaluation

Cell line–virus combinations were evaluated by infecting each cell line with HPIV-3, HSV-1, Ad5 and Reo-3 at multiple viral concentrations. Each experimental condition was performed in triplicate. Infection kinetics, including onset time and progression of CPE, were compared across all tested combinations to assess sensitivity and suitability for viral detection assays.

### 2.7. Data Analysis

Data were analyzed descriptively. Comparative analysis of infection kinetics and CPE development across different cell line–virus pairings was performed qualitatively and quantitatively based on observed time-to-CPE and LOQ values.

## 3. Results

### 3.1. Viral Propagation and Titers

All viruses used in this study were successfully propagated in permissive cell lines and reached comparable infectious titers. The final titers were determined as 1.3 × 10^10^ TCID_50_/mL for Ad5, 2.2 × 10^8^ TCID_50_/mL for Reo-3, 7.1 × 10^7^ TCID_50_/mL for HPIV-3 and 8.6 × 10^7^ TCID_50_/mL for HSV-1 (Table 1). These titers were sufficient to perform subsequent dilution-based infection assays and evaluate detection sensitivity across different cell line–virus combinations.

### 3.2. Cytopathic Effect (CPE) Kinetics Differ Across Cell Lines

Clear differences in cytopathic effect (CPE) kinetics were observed between the tested cell lines following infection with all viruses. The onset time, progression, and intensity of CPE varied depending on the cell line–virus combination.

#### 3.2.1. CPE Kinetics and Morphological Characteristics Induced by Reovirus Type 3 (Reo-3)

**Figure 1:**
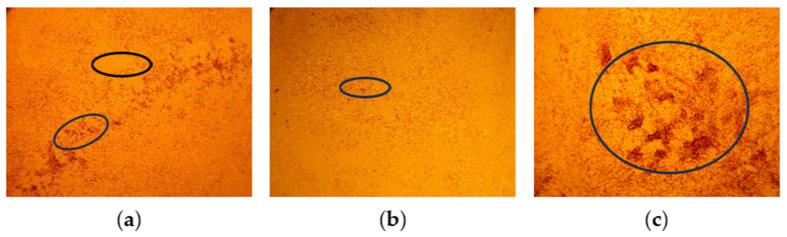
(**a**) Reo-3 in Vero cell line in titer 0.1 TCID_50_/mL; (**b**) Reo-3 in MRC-5 cell line in titer 0.1 TCID_50_/mL; (**c**) Reo-3 in BHK-21 [C-13] cell line in titer 0.1 TCID_50_/mL. Black circles indicate prominent cell rounding and monolayer detachment. Original magnification 40×.

Infection with Reovirus type 3 (Reo-3) revealed pronounced differences in cytopathic effect (CPE) kinetics across Vero, MRC-5, and BHK-21 [C-13] cell lines, with clear dependence on viral concentration (Figure 1).

In Vero cells, CPE developed rapidly and, in a concentration-dependent manner. At 10 TCID_50_/mL, initial CPE was observed at 24 h post-infection, with complete monolayer disruption by 72 h. At 1 TCID_50_/mL, onset occurred at 36 h, with full CPE also reached by 72 h. At 0.1 TCID_50_/mL, initial CPE appeared at 48 h and progressed to completion by day 4, while at 0.01 TCID_50_/mL, onset was observed at 72 h with full CPE by day 6. Infection was characterized by pronounced cell rounding, progressive detachment from the substrate, and the formation of well-defined focal areas of infection. The boundaries of these foci were clearly distinguishable, enabling straightforward visual identification of infected regions.

MRC-5 cells exhibited slower and more gradual infection kinetics. At 10 TCID_50_/mL, initial CPE was detected at 48 h and reached completion by day 4. At 1 TCID_50_/mL, onset occurred at 72 h with full CPE by day 5. At 0.1 TCID_50_/mL, initial changes were observed at day 5 and progressed to full CPE by day 7. At 0.01 TCID_50_/mL, initial CPE appeared at day 6, with complete monolayer disruption only by day 9. CPE developed more gradually and exhibited a less defined morphology. Infected cultures showed diffuse areas of monolayer degradation with slower cell rounding and less distinct borders between affected and unaffected regions. This resulted in a more heterogeneous appearance of the monolayer, particularly at intermediate stages of infection.

In contrast, BHK-21 [C-13] cells demonstrated the most rapid CPE progression among all tested cell lines. At 10 TCID_50_/mL, initial CPE was observed at 24 h, with complete CPE achieved by 48 h. At 1 TCID_50_/mL, onset occurred at 36 h and progressed to full CPE by day 3. At 0.1 TCID_50_/mL, initial changes were detected at 48 h, reaching completion by day 4. Even at the lowest concentration (0.01 TCID_50_/mL), CPE was observed relatively early at day 3, with full monolayer disruption achieved by day 5. BHK-21 [C-13] cells displayed rapid and highly pronounced morphological changes. Infection led to extensive cell rounding, rapid loss of adhesion, and the formation of distinct gaps within the monolayer. The resulting CPE was sharp, uniform, and easily distinguishable from non-infected areas, making it the most visually unambiguous among the tested cell lines.

Overall, BHK-21 [C-13] cells showed the fastest and most aggressive CPE kinetics for Reo-3, followed by Vero cells, while MRC-5 cells exhibited the slowest and most gradual progression. Observations indicate that, beyond differences in infection kinetics, the morphology of CPE varies significantly between cell lines and may influence the ease and reliability of visual interpretation in viral detection assays.

#### 3.2.2. CPE Kinetics and Morphological Characteristics Induced by Adenovirus Type 5 (Ad5)

**Figure 2:**
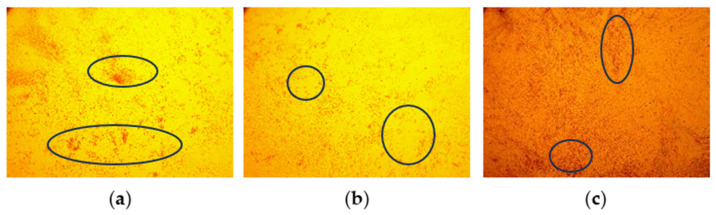
(**a**) Ad5 in Vero cell line in titer 0.1 TCID_50_/mL; (**b**) Ad5 in MRC-5 cell line in titer 0.1 TCID_50_/mL; (**c**) Ad5 in BHK-21 [C-13] cell line in titer 0.1 TCID_50_/mL. Black circles indicate prominent cell rounding and monolayer detachment. Original magnification 40×.

In MRC-5 cells, CPE development followed a consistent, concentration-dependent pattern (Figure 2). At 10 TCID_50_/mL, initial CPE was observed at 72 h, reaching full development by day 4. At 1 TCID_50_/mL, onset occurred at 96 h with complete CPE by day 5. At 0.1 TCID_50_/mL, initial changes were observed at day 4 and progressed to full CPE by day 6. The lowest concentration (0.01 TCID_50_/mL) resulted in initial CPE at day 5 and complete monolayer disruption by day 7. Infection was associated with progressive cell rounding accompanied by the formation of clustered cell aggregates. These clusters appeared as compact groups of rounded cells, often maintaining partial attachment to the substrate during early stages of infection. Over time, the monolayer exhibited increasing structural disruption, although the process remained relatively gradual and spatially heterogeneous.

In BHK-21 [C-13] cells, Ad5 infection showed slower and less efficient progression. At 10 TCID_50_/mL, initial CPE appeared at 96 h and reached completion by day 6. At 1 TCID_50_/mL, onset was observed at day 5 with full CPE by day 7. At 0.1 TCID_50_/mL, initial CPE occurred at day 6 and progressed to full CPE by day 9. At the lowest concentration (0.01 TCID_50_/mL), initial CPE was observed at day 7; however, full CPE was not achieved in all replicates, indicating reduced sensitivity and reproducibility in this cell line. Morphological changes were less uniform compared to other cell lines. CPE was characterized by gradual and sometimes incomplete detachment of the monolayer. In several cases, only partial disruption of the cell layer was observed, with residual areas of adherent cells persisting even at later stages of infection. This resulted in less distinct and less consistent visual patterns of CPE, particularly at lower viral concentrations.

Vero cells demonstrated intermediate kinetics compared to the other models. At 10 TCID_50_/mL, initial CPE was observed at 72 h, with full CPE by day 4. At 1 TCID_50_/mL, onset occurred at 96 h and reached completion by day 5. At 0.1 TCID_50_/mL, initial CPE was observed at day 4 with full development by day 6. At 0.01 TCID_50_/mL, initial changes appeared at day 5 and progressed to complete CPE by day 7. Infection induced characteristic cell rounding and the formation of localized cell clusters, followed by progressive detachment from the substrate. At later stages of infection, extensive gaps within the monolayer became evident, reflecting advanced cytopathic damage. The transition from early clustering to large areas of cell loss was gradual but clearly distinguishable, providing a relatively well-defined visual progression of infection.

Overall, MRC-5 and Vero cells provided more consistent and reproducible detection of Ad5 across the tested concentration range, whereas BHK-21 [C-13] cells exhibited reduced sensitivity at low viral concentrations. These findings highlight the importance of cell line selection for reliable detection of adenoviral contamination, particularly near the lower limits of quantification.

#### 3.2.3. CPE Kinetics and Morphological Characteristics Induced by HPIV-3 Infection

In Vero cells, HPIV-3 induced a rapid and pronounced cytopathic response even at the lowest tested concentration (0.01 TCID_50_/mL). Initial CPE was observed as early as 24 h post-infection, with complete monolayer destruction reached by day 4. Morphologically, infection was characterized by prominent cell rounding, formation of gaps within the monolayer, and progressive detachment of cells from the substrate. These changes became increasingly widespread over time, resulting in extensive disruption of the cell layer.

In MRC-5 cells, CPE progression was more gradual and concentration-dependent. At 10 TCID_50_/mL, initial cytopathic changes were observed at 24 h, with full CPE achieved by 72 h. At 1 TCID_50_/mL, onset occurred at day 2, progressing to complete CPE by day 4. At lower concentrations (0.1 and 0.01 TCID_50_/mL), similar kinetics were observed, with initial CPE detected at day 3 and full monolayer disruption occurring between day 5 and day 6. Morphologically, infected cultures displayed marked cell rounding and progressive detachment from the substrate, accompanied by gradual loss of monolayer integrity.

BHK-21 [C-13] cells exhibited infection kinetics comparable to those observed in MRC-5 cells; however, additional morphological features were noted. While cell rounding and detachment were also present, the monolayer displayed characteristic formation of voids and empty spaces within the cell layer. These gaps became progressively more pronounced as infection advanced, contributing to a more heterogeneous pattern of monolayer degradation compared to MRC-5 cells.

**Figure 3:**
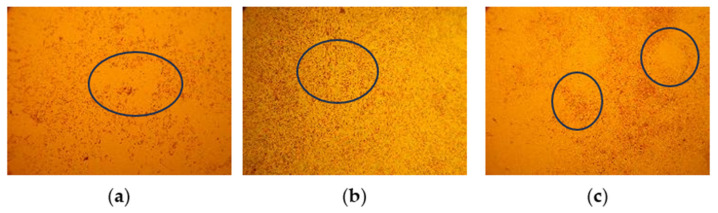
(**a**) HPIV-3 in Vero cell line in titer 0.1 TCID_50_/mL; (**b**) HPIV-3 in MRC-5 cell line in titer 0.1 TCID_50_/mL; (**c**) HPIV-3 in BHK-21 [C-13] cell line in titer 0.1 TCID_50_/mL. Black circles indicate prominent cell rounding and monolayer detachment. Original magnification 40×.

Overall, HPIV-3 demonstrated high infectivity across all tested cell lines, with Vero cells showing the fastest and most pronounced CPE, whereas MRC-5 and BHK-21 [C-13] cells exhibited more gradual progression (Figure 3). The additional formation of empty monolayer regions in BHK-21 [C-13] cells represents a distinguishing morphological feature that may influence visual interpretation in viral detection assays.

#### 3.2.4. CPE Kinetics and Morphological Characteristics Induced by HSV-1 Infection

In Vero cells, HSV-1 induced a rapid cytopathic response characterized by strong dose dependence. At the highest concentration (10 TCID_50_/mL), high levels of CPE were observed as early as 36 h post-infection. At 1 TCID_50_/mL, complete cytopathic effect was reached by day 3. At lower concentrations (0.1 and 0.01 TCID_50_/mL), infection kinetics were slower but remained highly consistent, with full CPE observed within 4 days, similarly to that observed for HPIV-3 in MRC-5 cells. Morphologically, infected cultures exhibited pronounced cell rounding, detachment from the substrate, formation of dense cellular aggregates, and progressive development of gaps within the monolayer.

In MRC-5 cells, HSV-1 infection progressed in a concentration-dependent manner with slightly delayed kinetics compared to Vero cells. At 10 TCID_50_/mL, complete CPE was observed by day 3. At 1 TCID_50_/mL, initial CPE was detected at 36 h, progressing to full monolayer destruction by day 4. At 0.1 TCID_50_/mL, initial cytopathic changes appeared at 48 h, with complete CPE achieved by day 5. At the lowest concentration (0.01 TCID_50_/mL), full CPE was observed by day 6. Morphological changes included cell rounding and progressive disruption of monolayer integrity, accompanied by partial detachment and loss of intercellular adhesion, resulting in areas of monolayer rupture.

In BHK-21 [C-13] cells, HSV-1 exhibited the most rapid and aggressive cytopathic progression among all tested conditions. Even at the lowest viral concentration (0.01 TCID_50_/mL), complete CPE was achieved within 2 days post-infection, leading to total monolayer degradation. The infection was characterized by extensive and uniform cell rounding, rapid loss of adherence, and complete collapse of cellular architecture, with minimal preservation of intact monolayer regions.

**Figure 4:**
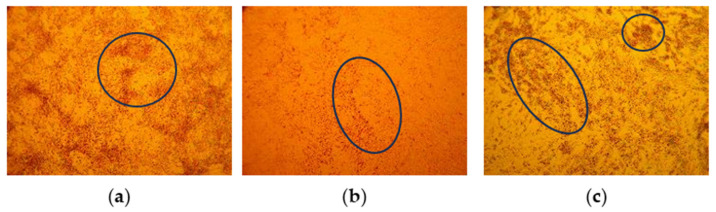
(**a**) HSV-1 in Vero cell line in titer 0.1 TCID_50_/mL; (**b**) HSV-1 in MRC-5 cell line in titer 0.1 TCID_50_/mL; (**c**) HSV-1 in BHK-21 [C-13] cell line in titer 0.1 TCID_50_/mL. Black circles indicate prominent cell rounding and monolayer detachment. Original magnification 40×.

Overall, HSV-1 demonstrated high infectivity across all tested cell lines(Figure 4), with Vero and BHK-21 [C-13] cells showing particularly rapid and pronounced cytopathic responses. MRC-5 cells exhibited more gradual progression, allowing for better temporal resolution of infection stages. The exceptionally fast and destructive CPE observed in BHK-21 [C-13] cells suggests high sensitivity but limited suitability for assays requiring controlled observation windows.

A summary of CPE kinetics across all tested conditions is presented in Table 2.

To facilitate comparative interpretation, cell lines were ranked based on detection sensitivity, CPE kinetics, and morphological clarity (Table 3). The ranking is based on comparative experimental observations and should be interpreted as a qualitative decision-support tool rather than a strictly quantitative metric.

## 4. Discussion

The present study systematically evaluated selected cell line–virus combinations to identify optimal systems for sensitive and reproducible viral detection assays in biopharmaceutical testing. The results clearly demonstrate that assay performance depends not only on analytical sensitivity, but also on cytopathic effect (CPE) kinetics, morphology, and interpretability.

Across all tested models, substantial variability was observed between cell lines in terms of infection dynamics and morphological presentation. While Vero and MRC-5 cells generally provided balanced and reproducible responses, BHK-21 [C-13] cells exhibited the most rapid and pronounced cytopathic effects, particularly for highly permissive viruses. This highlights an important methodological consideration: maximal sensitivity does not necessarily correspond to optimal assay performance, especially in systems requiring controlled observation windows.

Reovirus type 3 (Reo-3) showed the most distinct and rapid cytopathic response in BHK-21 [C-13] cells. Compared to Vero and MRC-5, this cell line enabled the earliest detection of infection across all tested concentrations, including low viral titers. Importantly, the CPE in BHK-21 [C-13] was highly pronounced and morphologically unambiguous, characterized by rapid cell rounding, loss of adhesion, and clear disruption of the monolayer. Although the accelerated kinetics may limit their use in long-term kinetic studies, these features make BHK-21 [C-13] particularly suitable for rapid screening assays where early detection and clear visual endpoints are prioritized.

For Adenovirus type 5 (Ad5), Vero cells provided the most consistent and interpretable results. In comparison to BHK-21 [C-13], where incomplete or variable CPE was observed at lower concentrations, Vero cells demonstrated reproducible infection kinetics and clear morphological progression across the full concentration range. The combination of moderate CPE development rate and well-defined structural changes supports the use of Vero cells as a reliable system for adenoviral detection, particularly in applications requiring both sensitivity and assay robustness.

Human parainfluenza virus type 3 (HPIV-3) exhibited high infectivity across all tested cell lines; however, MRC-5 cells provided the most balanced performance. While Vero cells showed very rapid CPE development, the slower and more controlled progression observed in MRC-5 allowed for improved temporal resolution and more consistent interpretation, particularly at lower viral concentrations. Morphological features such as gradual cell rounding and detachment further supported reliable visual assessment, making this system well-suited for standardized detection assays.

A similar pattern was observed for HSV-1, where MRC-5 cells enabled reproducible and concentration-dependent CPE progression. Although Vero and BHK-21 [C-13] cells exhibited faster cytopathic responses, the more controlled kinetics in MRC-5 provided a clearer distinction between infection stages. This is particularly important in quality control environments, where reproducibility and interpretability are critical. The observed morphology, including progressive monolayer disruption and cell rounding, further supports the suitability of this system for routine viral detection.

Importantly, despite comparable limits of quantification (LOQ) across multiple systems, the present study demonstrates that LOQ alone is insufficient for selecting optimal detection models. Instead, assay performance must be evaluated as a combination of sensitivity, CPE kinetics, and morphological clarity.

Based on these considerations, the following cell line–virus pairs were identified as optimal for different assay purposes:

- BHK-21 [C-13]/Reo-3—optimal for rapid and highly sensitive detection, with clear and early CPE development
- Vero/Ad5—providing robust and reproducible detection with well-defined cytopathic progression.
- MRC-5/HPIV-3—offering controlled kinetics and reliable interpretation across a wide concentration range.
- MRC-5/HSV-1—enabling reproducible and temporally resolved detection suitable for standardized assays.

These combinations reflect a balance between analytical sensitivity and practical assay performance, highlighting the importance of tailoring detection systems to specific viral characteristics and intended applications.

From a practical perspective, the results of this study are directly relevant to the design of viral safety testing strategies in biopharmaceutical production. According to regulatory guidelines such as ICH Q5A, the use of appropriate cell-based assays is a critical component of viral contamination risk assessment.

However, a significant limitation of this study is its reliance on visual assessment of cytopathic effect (CPE) as the primary endpoint. Although this is a very practical approach in routine quality control testing of biopharmaceutical products, morphological changes such as cell rounding, detachment, and disruption of the monolayer are not inherently virus-specific. These phenotypes largely overlap with general host cell damage, culture stress, or cellular remodeling induced by signaling pathways. For example, recent studies on RBMX2-associated infections have shown that pathogen interactions can profoundly reshape host epithelial biology, triggering intracellular pathways associated with apoptosis, morphological changes similar to EMT, and altered cell adhesion [11,12]. Although these studies do not focus specifically on the investigation of random viruses, they highlight an important biological principle: the observed cytopathic patterns may reflect host-directed response pathways rather than solely direct, productive viral replication. Therefore, the LOQ values reported in this study should be interpreted as operational thresholds for visual screening under standard conditions, rather than as strictly validated analytical limits of quantification. Furthermore, in industrial quality control, an adventitious viral contaminant might originate from a different host–cell system than the one used for in vitro testing. This host-matrix divergence can profoundly influence infection kinetics; for instance, an Adenovirus 5 strain propagated efficiently in human Hep2 cells might experience an adaptation barrier when introduced to simian Vero cells, leading to a prolonged eclipse phase and delayed CPE development. This principle is valid in general for any cross-species viral contamination event. To mitigate the risk of false-negative results during the window of viral adaptation, we recommend a robust dual-testing approach for industrial quality control. Utilizing a susceptible cell culture model for broad visual screening should ideally be complemented by confirmatory, highly sensitive molecular methods, such as virus-specific qPCR or next-generation sequencing (NGS), ensuring the most reliable and rapid pathogen detection.

The findings presented here support a rational approach to cell line selection, where the choice of indicator cells can be tailored depending on assay priorities, such as sensitivity, speed, or interpretability.

Importantly, the results suggest that no single cell line is universally optimal and that a combination of complementary models may provide a more robust detection strategy, particularly in high-risk quality control settings.

## 5. Conclusions

This study demonstrates that the selection of appropriate cell line–virus pairs is a critical determinant of assay performance in viral detection systems used for biopharmaceutical testing. While high analytical sensitivity was observed across multiple models, significant differences in cytopathic effect kinetics and morphology highlight the importance of considering additional parameters beyond the limit of quantification.

The results show that highly permissive cell lines, such as BHK-21 [C-13], can provide rapid and unambiguous detection for specific viruses, as demonstrated for Reovirus type 3. In contrast, more stable systems, such as MRC-5, offer improved reproducibility and temporal control, which are essential for standardized assays. Vero cells represent a balanced model, combining sensitivity with clear and interpretable cytopathic progression, particularly for Adenovirus type 5.

Based on a comprehensive evaluation of sensitivity, infection kinetics, and morphological clarity, the following cell line–virus pairs were identified as optimal: BHK-21 [C-13]/Reovirus type 3, Vero/Adenovirus type 5, and MRC-5/Human parainfluenza virus type 3 and Herpes simplex virus. These combinations provide a practical balance between rapid detection and assay robustness.

Overall, the study highlights the need for a rational and application-driven approach to assay design. The presented findings support the development of standardized and reliable viral detection strategies aligned with regulatory expectations and applicable to routine quality control in biopharmaceutical manufacturing.

## Figures and Tables

**Table 1:** Tested viruses with measured titers.

Virus Name	Ad5	Reo-3	HPIV-3	HSV-1
Titer [TCID_50_/mL]	1.3 × 10^10^	2.2 × 10^8^	7.1 × 10^7^	8.6 × 10^7^

**Table 2:** Summary of time-to-CPE and progression dynamics across cell line–virus combinations at different viral concentrations.

Virus Name	Cell Line	Concentration [TCID_50_/mL]	Time to Initial CPE	Time to Full CPE	Observed CPE Characteristic
Reo-3	Vero	10	24 h	72 h	Clear foci, cell rounding, detachment
Reo-3	Vero	1	36 h	72 h	Well-defined focal infection areas
Reo-3	Vero	0.1	48 h	Day 4	Progressive monolayer disruption
Reo-3	Vero	0.01	72 h	Day 6	Distinct infection foci, slower spread
Reo-3	MRC-5	10	48 h	Day 4	Diffuse monolayer degradation
Reo-3	MRC-5	1	72 h	Day 5	Gradual CPE, less defined borders
Reo-3	MRC-5	0.1	Day 5	Day 7	Heterogeneous monolayer damage
Reo-3	MRC-5	0.01	Day 6	Day 9	Slow progression, diffuse morphology
Reo-3	BHK-21	10	24 h	48 h	Rapid, uniform CPE, strong detachment
Reo-3	BHK-21	1	36 h	Day 3	Sharp and homogeneous CPE
Reo-3	BHK-21	0.1	48 h	Day 4	Clear monolayer gaps
Reo-3	BHK-21	0.01	Day 3	Day 5	Rapid and unambiguous CPE
Ad5	MRC-5	10	72 h	Day 4	Cell clustering, gradual disruption
Ad5	MRC-5	1	96 h	Day 5	Progressive aggregation and detachment
Ad5	MRC-5	0.1	Day 4	Day 6	Moderate, heterogeneous CPE
Ad5	MRC-5	0.01	Day 5	Day 7	Slower, consistent progression
Ad5	Vero	10	72 h	Day 4	Cluster formation, clear progression
Ad5	Vero	1	96 h	Day 5	Defined transition of infection stages
Ad5	Vero	0.1	Day 4	Day 6	Progressive cell loss
Ad5	Vero	0.01	Day 5	Day 7	Reproducible CPE pattern
Ad5	BHK-21	10	96 h	Day 6	Partial monolayer disruption
Ad5	BHK-21	1	Day 5	Day 7	Incomplete and variable CPE
Ad5	BHK-21	0.1	Day 6	Day 9	Weak and inconsistent morphology
Ad5	BHK-21	0.01	Day 7	Not complete	Low sensitivity, incomplete CPE
HPIV-3	Vero	0.01	24 h	Day 4	Rapid, strong CPE
HPIV-3	MRC-5	10	24 h	72 h	Gradual but consistent
HPIV-3	MRC-5	1	Day 2	Day 4	Controlled infection kinetics
HPIV-3	MRC-5	0.1	Day 3	Day 5–6	Reproducible progression
HPIV-3	MRC-5	0.01	Day 3	Day 5–6	Stable and interpretable
HPIV-3	BHK-21	0.01	Day 3	~Day 5	Formation of voids in monolayer
HSV-1	Vero	10	36 h	~Day 2–3	Rapid, dose-dependent CPE
HSV-1	Vero	1	~Day 2	Day 3	Strong morphological changes
HSV-1	Vero	0.1	~Day 2–3	Day 4	Consistent progression
HSV-1	Vero	0.01	~Day 3	Day 4	Reproducible kinetics
HSV-1	MRC-5	10	<48 h	Day 3	Gradual progression
HSV-1	MRC-5	1	36 h	Day 4	Controlled infection
HSV-1	MRC-5	0.1	48 h	Day 5	Progressive disruption
HSV-1	MRC-5	0.01	~Day 3–4	Day 6	Slower but consistent
HSV-1	BHK-21	0.01	<48 h	Day 2	Extremely rapid, destructive CPE

**Table 3:** Comparative ranking of cell lines based on detection sensitivity, CPE kinetics, and morphological clarity across tested viruses.

Virus Name	Cell Line	Detection Sensitivity	CPE Onset Speed	Morphological Clarity
Reo-3	BHK-21 [C-13]	High	Very fast	High
	Vero	High	Fast	High
	MRC-5	Moderate	Slow	Moderate
Ad5	MRC-5	High	Moderate	High
	Vero	High	Moderate	High
	BHK-21 [C-13]	Low	Slow	Low
HPIV-3	Vero	High	Very fast	High
	MRC-5	High	Moderate	High
	BHK-21 [C-13]	Moderate	Moderate	Moderate
HSV-1	BHK-21 [C-13]	High	Very fast	High
	Vero	High	Fast	High
	MRC-5	Moderate	Moderate	Moderate

Detection sensitivity was classified based on the lowest viral concentration at which reproducible CPE was observed. CPE onset speed was categorized as: very fast (<48 h), fast (48–72 h), moderate (3–5 days), or slow (>5 days). Morphological clarity reflects the ease of visual interpretation of CPE, including distinctness and reproducibility of structural changes.

## Data Availability

The original contributions presented in this study are included in the article. Further inquiries can be directed to the corresponding author.

## Abbreviations

The following abbreviations are used in this manuscript:

CPE	Cytopathic effect
LOQ	Limit of Quantification
TCID_50_	Tissue Culture Infection Dose
Reo-3	Reovirus 3
Ad5	Adenovirus 5
HSV-1	Human Herpes virus 1
HPIV-3	Human Parainfluenza virus type 3
PFU	Plaque Forming Unit
EMEM	Eagle’s Minimum Essential Medium
FBS	Fetal Bovine Serum
CHO	Chinese Hamster Ovary
